# Evaluation of Factors Impacting Shelter Cats’ Personalities

**DOI:** 10.3390/life16010155

**Published:** 2026-01-17

**Authors:** Mihai Borzan, Christelle Digonnet, Emoke Pall, Anamaria Ioana Paștiu, Alexandra Tabaran

**Affiliations:** 1 Department of Animal Breeding and Food Safety, Faculty of Veterinary Medicine, University of Agricultural Sciences and Veterinary Medicine Cluj-Napoca, 400372 Cluj-Napoca, Romania; mihai.borzan@usamvcluj.ro (M.B.); cristelle.digonnet@student.usamvcluj.ro (C.D.); alexandra.tabaran@usamvcluj.ro (A.T.); 2 Department of Infectious Diseases, Faculty of Veterinary Medicine, University of Agricultural Sciences and Veterinary Medicine Cluj-Napoca, 400372 Cluj-Napoca, Romania; 3 Department of Genetics and Hereditary Diseases, Faculty of Veterinary Medicine, University of Agricultural Sciences and Veterinary Medicine Cluj-Napoca, 400372 Cluj-Napoca, Romania; oana.pastiu@usamvcluj.ro

**Keywords:** cat, personality, shelters, behavioral

## Abstract

Behavior-related factors represent a major cause of cat relinquishment to shelters, highlighting the need for reliable tools to support appropriate matching between cats and adopters. The present study applied the ASPCA^®^ Meet Your Match^®^ Feline-ality™ assessment to evaluate personality profiles of shelter cats and to examine factors associated with variation in personality expression across shelters. A total of 113 cats housed in six shelters in the south of France were assessed using a standardized behavioral protocol. Differences between shelters were evaluated using one-way ANOVA for behavioral scale scores, while associations between personality type and shelter affiliation, sex, coat color, and age were analyzed using χ^2^ tests of independence. Significant differences between shelters were observed for the majority of behavioral assessment items, as well as for composite valiance and independent–gregarious scale scores. Shelter affiliation was significantly associated with the distribution of Feline-ality™ personality types, indicating that personality profiles were not uniformly distributed across shelters. No statistically detectable association was found between personality type and sex. In contrast, significant associations were observed between personality type and both coat color category and age category, suggesting non-random variation in personality distribution across these factors. These findings indicate that shelter-related and individual factors are associated with variation in feline personality expression. While causal relationships cannot be inferred, the results underscore the importance of considering environmental context and population characteristics when interpreting shelter-based behavioral assessments. The Feline-ality™ framework appears to be a useful tool for characterizing personality variation in shelter cats and may support improved adoption matching when applied with appropriate caution.

## 1. Introduction

The domestic cat (*Felis catus*) is one of the most common companion animals worldwide and occupies an increasingly important role in human society [1,2]. Despite their popularity, cats are frequently relinquished to shelters, with behavior-related issues representing one of the primary reasons for abandonment. Shelter environments therefore play a critical role not only in animal welfare but also in shaping behavioral expression and influencing adoption outcomes [3].

Previous research has shown that cats exhibit consistent individual differences in behavior that are commonly described as personality traits, including dimensions related to sociability, boldness, and responsiveness to human interaction [4,5,6]. However, the expression of these traits is not fixed and may vary depending on contextual factors such as age, prior experience, environmental conditions, and human–animal interactions [7,8,9,10]. In shelter settings, where animals are exposed to unfamiliar environments and varying levels of stress, assessing personality presents methodological challenges [11].

To address the high rate of post-adoption returns associated with behavioral mismatches, the American Society for the Prevention of Cruelty to Animals (ASPCA^®^) developed the Meet Your Match^®^ adoption program, which incorporates the Feline-ality™ behavioral assessment [12]. This standardized tool aims to characterize key dimensions of feline behavior and to support adoption matching by aligning cat personality profiles with adopter expectations. Although the Feline-ality™ framework has been validated and widely implemented in shelters, relatively few studies have examined how personality distributions assessed using this tool vary across shelters or how individual and environmental factors are associated with these distributions.

Shelter environments differ in numerous respects, including housing configuration, intake composition (e.g., feral versus previously owned cats), frequency of human interaction, and management practices. These factors may contribute to variation in observed personality profiles, particularly when assessments are conducted over relatively short time frames. Moreover, individual characteristics such as age, sex, and phenotypic traits (e.g., coat color) have been inconsistently associated with behavioral variation in cats, with previous studies reporting mixed and sometimes contradictory findings [6,13,14].

The present study applies the Feline-ality™ assessment to a population of shelter cats housed in six shelters in the south of France. Rather than testing causal hypotheses, this study adopts an observational approach to examine associations between shelter affiliation, individual characteristics, and the distribution of personality types. Specifically, the objectives were to (i) compare behavioral scale scores and personality distributions across shelters, and (ii) assess associations between personality type and selected individual factors, including sex, age, and coat color category. By clarifying patterns of variation within and between shelters, this study seeks to contribute to a more nuanced understanding of feline personality expression in shelter contexts and to inform the appropriate interpretation of shelter-based behavioral assessments.

## 2. Materials and Methods

### 2.1. Study Design and Subjects

**Figure 1:**
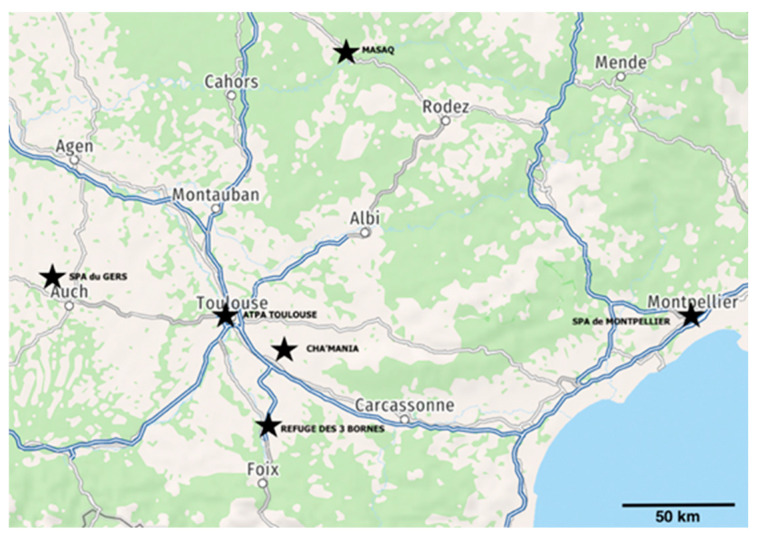
Geographical distribution of the six shelters included in the study (South of France). *The map was created by the authors based on publicly available geographic data*.

The study was conducted in collaboration with six animal shelters located in the South of France (Figure 1): Cha’Mania, SPA Montpellier, SPA du Gers, ATPA Toulouse, MASAQ, and Refuge des 3 Bornes. These shelters were distributed within a radius of approximately 160 km and were visited between January and April 2023. The number of assessment sessions and cats evaluated per shelter varied due to logistical constraints and shelter availability.

A total of 113 cats were included in the study, comprising 60 males and 53 females, aged between 9 months and 13 years. Cats originated either as strays or relinquished animals, and detailed background information prior to shelter admission was generally unavailable. All cats had been housed in the shelter for a minimum period required by local vaccination and sterilization protocols and were clinically healthy at the time of assessment. Pregnant queens and cats in estrus were excluded.). Nutritional information and specific prophylactic measures are detailed in Table 1.

Basic individual data were recorded for each cat, including sex, age, coat color category, and breed when known. Age was categorized into four groups for analysis: kittens (<1 year), young adults (1–6 years), mature adults (7–10 years), and seniors (>10 years).

### 2.2. Meet Your Match^®^ Program Application

Behavioral assessment was based on the Meet Your Match^®^ Program developed by the American Society for the Prevention of Cruelty to Animals (ASPCA), using the Feline-ality™ assessment tool. The objective of the present study was not to validate the Feline-ality™ tool, as its validity has been previously established, but to apply it comparatively across multiple shelters.

The Feline-ality™ assessment consists of a standardized series of behavioral observations conducted during a single session lasting approximately 20–25 min. The assessment includes 11 behavioral items evaluating posture, exploratory behavior, sociability, response to human interaction, and playfulness. Each item contributes points to one or both of two behavioral dimensions: the independent–gregarious scale and the valiance scale.

Total scores for each scale were calculated by summing item-level scores according to the Meet Your Match^®^ guidelines. Based on these composite scores, cats were classified into one of nine predefined Feline-ality™ personality types. All assessments were conducted by the same trained evaluator to minimize inter-observer variability.

To account for structural differences between French and American shelters, minor modifications to the first four assessment items were implemented following the protocol described by Fukimoto et al. (2019) [15]. All other components of the assessment adhered strictly to the original Meet Your Match^®^ guidelines.

### 2.3. Environmental Context

Although efforts were made to conduct assessments under comparable conditions, shelters differed in several contextual aspects, including housing configuration, daily routines, and frequency of human–cat interaction. These variables were not experimentally controlled and were therefore treated as inherent characteristics of each shelter rather than independent predictors.

### 2.4. Statistical Analysis

Statistical analyses were performed to evaluate both differences in behavioral outcomes between shelters and associations between personality type and individual cat characteristics. All analyses were conducted using standard statistical software, and the significance threshold was set at *p* ≤ 0.05.

#### 2.4.1. Analysis of Behavioral Items and Scale Scores

Differences between shelters for individual Feline-ality™ assessment items and for the composite valiance and independent–gregarious scale scores were analyzed using one-way analysis of variance (ANOVA). Shelter affiliation was treated as the independent factor (six levels), and item scores or scale scores were treated as dependent variables. When statistically significant effects were observed, effect sizes were quantified using eta squared (η^2^) to estimate the proportion of variance explained by shelter affiliation.

#### 2.4.2. Analysis of Personality Distribution

To examine whether the distribution of Feline-ality™ personality types differed across shelters, a χ^2^ test of independence was performed. This analysis tested the association between shelter affiliation (six categories) and personality type, using observed frequencies of cats classified into each personality category. Effect size for significant χ^2^ tests was estimated using Cramer’s V.

#### 2.4.3. Analysis of Individual Factors

Additional χ^2^ tests of independence were conducted to assess associations between Feline-ality™ personality type and individual characteristics, including sex, coat color category, and age category. Coat color was grouped into predefined categories (single-color, bi-color, tabby, and other patterns) to ensure sufficient expected cell counts. Age was analyzed using the four predefined age categories.

For all χ^2^ analyses, inferential conclusions were restricted to the presence or absence of statistical associations. Statements regarding the most frequently observed personality types within specific groups were based solely on descriptive frequency analyses and were not interpreted as inferential outcomes.

## 3. Results

The present study applied the Feline-ality™ assessment framework to evaluate personality profiles of 113 cats across six shelters. All statistical analyses were conducted to examine differences in personality distribution between shelters, as well as associations between personality type and individual cat characteristics, including sex, coat color, and age.

Analysis of the Feline-ality™ assessment items revealed statistically significant differences between shelters for the majority of behavioral variables. Specifically, 10 out of the 12 assessed items (items #2, #3, #4, #5a, #5b, #7, #8, #9, #10, and #11) differed significantly across shelters (one-way ANOVA, *p* < 0.05), indicating variability in cats’ sociability, valiance, and interaction patterns depending on shelter affiliation. In contrast, items #1 (body posture at approach) and #6 did not show statistically detectable differences between shelters.

One-way ANOVA analyses conducted on the composite Feline-ality™ scale scores demonstrated significant differences between shelters for both behavioral dimensions. Mean valiance scores differed significantly across shelters, F (5, 107) = 5.41, *p* < 0.001, with a large effect size (η^2^ = 0.20). Similarly, mean independent–gregarious scores also varied significantly between shelters, F (5, 107) = 3.77, *p* = 0.003, with a moderate effect size (η^2^ = 0.15). These results indicate that shelter affiliation accounted for a meaningful proportion of the variability observed in both dimensions of feline personality.

**Figure 2:**
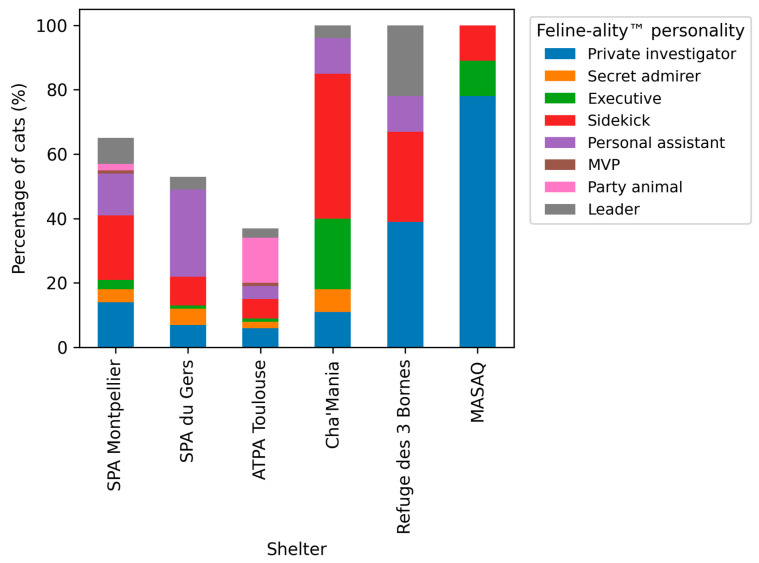
Distribution of Feline-ality™ personality types across the six shelters included in the study.

To further examine whether the distribution of personality types differed across shelters, a χ^2^ test of independence was performed to assess the association between shelter affiliation (six shelters) and Feline-ality™ personality type. The analysis revealed a statistically significant association, χ^2^(25, *n* = 113) = 47.62, *p* = 0.010. The strength of this association was moderate (Cramer’s V = 0.46), indicating that personality distributions were not uniform across shelters (Figure 2). The figure illustrates the relative frequencies of personality types within each shelter. Differences in distribution were evaluated using a χ^2^ test of independence, indicating a statistically significant association between shelter affiliation and personality type.

Overall, the findings underscore the importance of considering shelter-specific factors when assessing cat personalities and emphasize the need for standardized protocols to ensure consistency and comparability across shelters.

It is important to note that the χ^2^ test does not identify a predominant personality within a shelter but rather indicates that the distribution of personality types differs between shelters. Descriptive frequency analyses were therefore used to identify the most frequently observed personality types within each shelter, while inferential conclusions were limited to the presence of an association between shelter affiliation and personality type.

Descriptive analyses showed that shelters located in more rural contexts (MASAQ, Refuge des 3 Bornes, and Cha’Mania) exhibited higher frequencies of cats classified as Private Investigator, a personality profile associated with lower valiance and higher independence. In contrast, shelters characterized by higher levels of human–cat interaction (SPA Montpellier, SPA du Gers, and ATPA Toulouse) more frequently housed cats classified as Sidekick, reflecting moderate valiance and social tendencies. These descriptive patterns were consistent with the differences observed in both individual item scores and composite scale scores.

In addition to shelter-level comparisons, χ^2^ tests were conducted to explore potential associations between personality type and individual cat characteristics. No statistically detectable association was observed between personality type and sex (*p* > 0.05). In contrast, a significant association was found between coat color category and personality type, χ^2^ (21, n = 113) = 67.59, *p* < 0.001. This result indicates that the distribution of Feline-ality™ personality types differed across coat color categories in the present sample.

Finally, analysis of the relationship between personality type and age category revealed a statistically significant association, χ^2^ = 0.0273 (df (degrees of freedom), n = 113), *p* < 0.05. Younger cats (under one year of age) were more frequently classified into personality profiles characterized by higher independence and lower valiance, whereas adult and senior cats showed higher frequencies of social and higher-valiance personality types. These results suggest that age-related differences in personality distribution were present within the studied population.

## 4. Discussion

The present study investigated factors associated with personality variation in shelter cats using the Feline-ality™ assessment framework. The results demonstrate that shelter affiliation is significantly associated with differences in both behavioral scale scores (valiance and independent–gregarious) and the distribution of personality types, highlighting the importance of environmental and management-related factors in shaping feline behavior in shelter contexts [7,10].

Significant differences between shelters were observed for the majority of behavioral assessment items, as well as for composite valiance and independent–gregarious scale scores. These findings suggest that shelter-specific conditions contribute meaningfully to variation in feline personality expression. Although standardized assessment procedures were applied consistently across all shelters, cats were exposed to different environmental contexts, including variation in housing conditions, daily routines, and levels of human interaction. Previous studies have demonstrated that such environmental factors influence stress responses, exploratory behavior, and social interactions in cats housed in shelters [7,9,10,16,17].

The χ^2^ analysis further revealed a significant association between shelter affiliation and Feline-ality™ personality type. This result indicates that personality distributions were not uniform across shelters, but it does not imply that any single shelter deterministically produces a specific personality profile. Rather, this association reflects systematic differences in the populations housed within each shelter, likely shaped by intake sources (e.g., feral versus previously owned cats), length of stay, and frequency of interaction with caregivers and volunteers [10,12]. Descriptive analyses suggested that shelters with limited daily human–cat interaction more frequently housed cats classified as Private Investigator, whereas shelters characterized by more frequent human contact more often housed cats classified as Sidekick. These descriptive patterns are consistent with previous findings indicating that sociability and boldness in cats are influenced by environmental enrichment and human exposure [7,9].

In addition to shelter-level effects, the present study identified a significant association between coat color category and Feline-ality™ personality type. While this finding indicates non-random co-variation between coat color and personality classification, it should be interpreted with caution. Coat color itself is unlikely to be a direct determinant of feline personality. Instead, this association may reflect indirect relationships with other variables not explicitly controlled for in the present study, such as breed composition, genetic background linked to coat traits, or shelter-specific intake patterns. Previous studies investigating relationships between coat characteristics and behavior have reported mixed and sometimes contradictory results, underscoring the likelihood that any observed associations are context-dependent rather than causal [6,13,18].

With respect to individual characteristics, no statistically detectable association was observed between sex and personality type, a finding consistent with previous research indicating limited or no sex-related effects on feline personality traits [6,13]. In contrast, a significant association was found between age category and personality distribution. Younger cats were more frequently classified into personality types characterized by higher independence and lower valiance, whereas adult and senior cats more often exhibited social and higher-valiance profiles. This result suggests that personality expression in cats may change across the lifespan, potentially reflecting developmental processes, accumulated experiences, and adaptation to environmental demands. Similar age-related effects on behavior have been reported in both human and animal studies, indicating that personality traits may exhibit a degree of plasticity over time [11,17,18,19].

It is important to emphasize that geographical location per se cannot be interpreted as a causal determinant of feline personality in the present study. Cats were not randomly assigned to shelters, and multiple factors vary simultaneously across geographical locations. Therefore, differences observed between shelters are more appropriately attributed to factors which vary across the geographical locations studied, such as urban versus rural settings, socioeconomic context, local policies regarding free-roaming cats, and the proportion of feral versus previously owned animals [10,12]. These interacting factors may shape both shelter intake composition and the behavioral profiles observed.

### Limitations and Future Research

Several limitations of the present study should be acknowledged. First, the observational design and lack of random assignment of cats to shelters limit the ability to draw causal inferences regarding the determinants of personality variation [10]. Second, the number of cats assessed per shelter was unequal, which may have reduced statistical power for some comparisons and increased the risk of Type II error, particularly for analyses in which no statistically detectable association was observed. Third, although the Feline-ality™ assessment has been validated and was applied consistently by a single evaluator, the relatively short duration of the assessment may not capture the full complexity of feline personality, especially in animals experiencing shelter-related stress [12,15].

Additionally, several potentially influential environmental variables were not measured directly, including cage size and configuration, group versus single housing, ambient noise and light levels, feeding and cleaning schedules, and the presence of dogs within the shelter environment. Previous research has highlighted the influence of these factors on feline welfare and behavior in shelter settings, suggesting that their inclusion in future studies would improve explanatory power [7,9,16].

Future research should consider longitudinal designs that assess personality stability over time, including post-adoption follow-up to evaluate the predictive validity of shelter-based assessments such as Feline-ality™ [12,15]. Expanding sample sizes and including shelters from diverse geographic and cultural contexts would further enhance generalizability. Finally, integrating behavioral assessments with physiological measures of stress may provide a more comprehensive understanding of how shelter environments influence feline welfare and personality expression [7,10].

## 5. Conclusions

The present study demonstrates that feline personality profiles assessed using the Feline-ality™ framework vary significantly across shelters and are associated with both shelter affiliation and selected individual characteristics. Differences in valiance and sociability-related dimensions between shelters highlight the influence of shelter-level factors, such as environmental context and patterns of human–cat interaction, on behavioral expression in cats.

Associations between personality type and age suggest that personality expression may change across the lifespan, reflecting behavioral plasticity and accumulated experience. The observed association between coat color category and personality type indicates non-random co-variation within the studied population but should not be interpreted as evidence of a direct causal relationship. No association between sex and personality type was detected.

Importantly, the findings do not support the interpretation that geographical location alone determines feline personality. Rather, the results point toward factors that vary across geographical locations—such as shelter management practices, intake composition, and environmental exposure—as more plausible contributors to observed differences in personality distribution.

Overall, the Feline-ality™ assessment provides a structured and practical approach for characterizing personality variation in shelter cats. When interpreted within the context of environmental and methodological limitations, its application may contribute to more informed adoption decisions and improved welfare outcomes. Further research incorporating longitudinal designs and post-adoption follow-up is warranted to better understand the stability and predictive value of shelter-based personality assessments.

## Figures and Tables

**Table 1:** Details regarding food provision, sterilization, and vaccination protocols in the evaluated shelters.

Shelter	Nutrition	Sterilisation & Vaccination Protocol	Veterinarians
Cha’Mania	Unknown but dry food at will	All shelters had almost the same protocol: 1. Quarantine of 8 days (allow to check if apparition of clinical signs + time for potential owner to call for their pet) 2. Test FIV/FeLV 3. Anti-parasitic treatments 4. Vaccination against Panleukopenia, Feline Leukemia virus & Herpesvirus+Calicivirus 5. Booster vaccination 21 days after 6. Sterilisation 7. Identification 8. Cattery	Unkown
SPA Montpellier	Royal Canin Neutered Adult Dry food at will		1 sanitary veterinarian Other veterinarians doing vaccinations & sterilisation (number not known)
SPA du Gers	Premiere adult & junior Dry food at will		1 sanitary veterinarian 2 vet clinics
ATPA Toulouse	Unknown but dry food at will		Unkown
Refuge des 3 Bornes	Chicken Ownat Adult Dry food at will		1 sanitary veterinarian Vet clinic with 7 vets
MASAQ	Unknown but dry food at will		Unkown

## Data Availability

The data presented in this study are available on request from the corresponding author due to privacy.

## References

[1] Vigne J.D., Guilaine J., Debue K., Haye L., Gérard P. (2004). Early taming of the cat in Cyprus. Science.

[2] Cameron-Beaumont C., Lowe S.E., Bradshaw J.W.S. (2002). Evidence suggesting preadaptation to domestication throughout the small Felidae. Biol. J. Linn. Soc..

[3] Edwards C., Heiblum M., Tejeda A., Galindo F. (2007). Experimental Evaluation of Attachment Behaviors in Owned Cats. J. Vet. Behav..

[4] Rochlitz I., Yeates J., Yeates J. (2018). Cats (*Felis silvestris catus*). Companion Animal Care and Welfare.

[5] Bernstein P.L., Turner D.C., Bateson P. (2007). The human–cat relationship. The Welfare of Cats.

[6] Salonen M., Vapalahti K., Tiira K., Mäki-Tanila A., Lohi H. (2019). Breed differences of heritable behaviour traits in cats. Sci. Rep..

[7] Stella J., Croney C., Buffington T. (2013). Effects of stressors on the behavior and physiology of domestic cats. Appl. Anim. Behav. Sci..

[8] Zasloff R.L. (1996). Measuring Attachment to Companion Animals: A Dog Is Not a Cat Is Not a Bird. Appl. Anim. Behav. Sci..

[9] Adamelli S., Marinelli L., Normando S., Bono G. (2005). Owner and Cat Features Influence the Quality of Life of the Cat. Appl. Anim. Behav. Sci..

[10] Powell L., Reinhard C.L., Satriale D., Morris M., Serpell J., Watson B. (2022). The Impact of Returning a Pet to the Shelter on Future Animal Adoptions. Sci. Rep..

[11] Specht J., Egloff B., Schmukle S.C. (2011). Stability and change of personality across the life course: The impact of age and major life events on mean-level and rank-order stability of the Big Five. J. Pers. Soc. Psychol..

[12] American Society for the Prevention of Cruelty to Animals® (ASPCA). Meet Your Match Pet Adoption Program. https://www.aspcapro.org/resource/meet-your-match-pet-adoption-program.

[13] Wilhelmy J., Serpell J., Brown D., Siracusa C. (2016). Behavioral Associations with Breed, Coat Type, and Eye Color in Single-Breed Cats. J. Vet. Behav..

[14] Kaleta T., Borkowska N., Góral-Radziszewska K. (2016). The Study of Domestic Cat (*Felis catus*) Personality Based on Survey in Poland. Anim. Sci..

[15] Fukimoto N., Howat-Rodrigues A.B., Mendonça-Furtado O. (2019). Modified Meet Your Match^®^ Feline-Ality^TM^ Validity Assessment: An Exploratory Factor Analysis of a Sample of Domestic Cats in a Brazilian Shelter. Appl. Anim. Behav. Sci..

[16] Mauro I., Ricci-Bonot C., Mills D.S. (2021). My Cat and Me—A Study of Cat Owner Perceptions of Their Bond and Relationship. Animals.

[17] Howell T.J., Bowen J., Fatjó J., Calvo P., Holloway A., Bennett P.C. (2017). Development of the Cat-Owner Relationship Scale (CORS). Behav. Process..

[18] González-Ramírez M.T., Landero-Hernández R. (2022). Cat Coat Color, Personality Traits and the Cat-Owner Relationship Scale: A Study with Cat Owners in Mexico. Animals.

[19] Delgado M.M., Munera J.D., Reevy G.M. (2012). Human perceptions of coat color as an indicator of domestic cat personality. Anthrozoös.

